# The impact of juvenile hypothyroidism on stature

**DOI:** 10.25122/jml-2022-0303

**Published:** 2023-08

**Authors:** Alyaa Farouk Al-Omari, Zayd kays Omer

**Affiliations:** 1 Department of Physiology, College of Medicine, Ninevah University, Mosul, Iraq; 2 Department of Physiology, College of Medicine, University of Mosul, Mosul, Iraq

**Keywords:** children, growth, hypothyroidism, juvenile, stature

## Abstract

Short stature with different alterations in the skeletal appearance usually results from juvenile hypothyroidism. The present case-control study was conducted to assess the effect of thyroid hormone deficiency on the height of young people and the prevalence of short stature in juvenile hypothyroidism. The research was conducted at the Al-Waffa Diabetic Centre between May and September 2022. The study group included 90 children with short stature, while the control group included 58 children. The statistical analysis was conducted using Minitab version 20. The results revealed that a low serum level of triiodothyronine (T3) was present in 2.2% of participants, while a low serum level of thyroxin (T4) was present in 36.7%. All subjects had elevated serum thyroid stimulating hormone (TSH). Female children had increased rates of short stature. Juvenile hypothyroidism results in various skeletal manifestations, including reduced height. Low serum thyroxin coupled with high serum thyroid stimulating hormone are common findings in juvenile hypothyroidism.

## INTRODUCTION

When a patient's linear growth does not reach the threshold of four cm per year (the limits in the case of children's growth changes are lower), they are considered to have short stature. A child or adolescent's height must be considerably below the third percentile or two standard deviations (SD) below the mean height for their age and gender to be considered of short stature. Endocrine and metabolic disorders, general systems disorders, uncontrolled diabetes with altered mineral levels, malnutrition, emotional illness, dysmorphic syndromes, congenital metabolic errors, and chromosomal abnormalities can inhibit linear growth and development [1-3].

A predicted adult height of 160cm for men and 150 cm for women is used to diagnose idiopathic short stature, which is defined for this purpose by a stricter height criterion (below -5.7 SD) [2, 4].

Thyroid hormones directly impact human growth and development and indirectly affect growth hormones. Therefore, juvenile hypothyroidism is generally characterized by short height. Reduced spontaneous growth hormone secretion and muted responses to growth hormone stimulating tests are manifestations of hypothyroidism [5].

The frequency of juvenile hypothyroidism is not well understood due to a lack of research. In underdeveloped nations with scarce access to healthcare, hypothyroidism is a key contributor to short stature. A study by Chowdhury *et al*. revealed that 28% of female participants and 19% of male participants with short stature had hypothyroidism [6].

Juvenile hypothyroidism is a common condition that is frequently ignored. It occurs when an autoimmune disease damages the thyroid gland, causing a lack of thyroid hormone in childhood or adolescence. Untreated congenital hypothyroidism is currently uncommon due to the existence of screening programs, and it typically manifests with delayed puberty, small stature, goiter, and irregular menstruation [7]. However, developing nations continue to see a high prevalence of juvenile hypothyroidism [8].

The mean age of diagnosis for patients with juvenile hypothyroidism is 11.2±2.3 years and male children outnumber female children by 2:1. The patients' presentations include low height, spondylolisthesis, delayed bone age, and abnormal epiphyseal ossification. Early identification of the abnormalities can result in prompt and efficient therapy, reducing the incidence of short stature and skeletal symptoms [9].

### Study aim

The present study was conducted to assess the effect of thyroid hormone deficiency on the height of children and the prevalence of short stature in juvenile hypothyroidism.

## MATERIAL AND METHODS

A case-control study was conducted at the Al-Waffa Diabetic Centre between May and September 2022. The sample consisted of 90 children with short stature (38 male children and 52 female children), with a mean age of 10.6±3.01 years. The boys' age ranged between 6-15 years whereas the girls' age ranged from 5-15 years. The control group was comprised of 58 children with normal height (34 male, 24 female), with a mean age of 11.0±2.91 years, (boys: 6-15 years; girls: 7-15 years).

The causes of short stature in children, specifically hypothyroidism, were explored. Anthropometry was one of the clinical and laboratory characteristics used to rule out any systemic chronic conditions. Serum thyroid function tests, such as triiodothyronine (T3), thyroxin (T4), and thyroid stimulating hormone (TSH) measurement by radioimmunoassay, were used to confirm the diagnosis, using bio Merieux's minividas kit, which is a commercially available kit. Standing height was measured with a Harpenden Stadiometer and represented as standard deviation score (SDS) for chronological age in accordance with Tanner *et al*. who used accurate measurements and age/sex-specific development charts [10]. Middle East growth norms were chosen because they are comparable to our regional percentiles [11].

### Statistical analysis

The statistical analysis was conducted using Minitab version 20. Continuous data were expressed using means and standard deviations, while categorical variables were presented as percentages. The Chi-square test was used to compare categorical parameters, while the Student’s t-test was employed to evaluate continuous parameters. Correlations were computed using the Pearson's test. The findings are presented as mean ±SD and the statistical significance was determined at a p-value≤0.05.

## RESULTS

The mean age for the short stature group was 10.6±3.01 years and for the control group was 11.0±2.91 years presenting no significant difference.

The between-group height comparison showcased a significant statistical difference (p<0.001), with a higher range of height in the control group. The mean ±SD was 143.3±13.45 cm compared to the short stature group (128.3±13.69 cm), as shown in Table 1.

**Table 1:** Personal characteristics

Parameters	Cases “Short stature” [N=90]		Control “Normal height” [N=58]		p-value*
	Mean ± SD	Range	Mean ± SD	Range	
**Age (years)**	10.6±3.01	5-15	11.0±2.91	6-15	0.431
**Height (cm)**	128.3±13.69	100-149	143.3±13.45	118-161	0.001
**Gender**	**No**.	**%**	**No**	**%**	p-value**
**Male**	38	42.2	34	58.6	0.051
**Female**	52	57.8	24	41.4	

* Independent T-test of two means was used for measurable variables; **Chi-square test applied for gender differences

By comparing male and female gender, it was revealed that short stature was less common in boys (N=38; 42.2%) than girls (N=52; 57.8%), with no significant difference, as presented in Table 1.

When comparing serum T3 level, the study group insignificantly differs from the control group, as shown in Table 2.

**Table 2:** Serum levels of T3

T3 level	Cases “Short stature”		Control “Normal height”		Odd ratio	95% C.I.	p-value
	No.	%	No.	%			
**Low** **[<1.2 mmol/l]**	2	2.2	2	3.5	0.64	0.09; 4.65	0.653*
**Normal and high** **[≥1.2 mmol/l]**	88	97.8	56	96.5			
**Total**	90	100	58	100	---	---	---
**Mean ± SD**	2.47±1.12		2.41±0.69		---		0.725**

* Chi-square test applied for gender differences; **Independent T-test of two means was used for measurable variables

Table 3 shows that the mean of serum T4 level in short stature group (68.1±18.7 mmol/l) is significantly (p<0.001) lower than that of normal height group (129.2±32.6 mmol/l).

**Table 3:** Serum levels of T4

T4 level	Cases “Short stature”		Control “Normal height”		Odd ratio	95% C.I.	p-value
	No.	%	No.	%			
**Low** **[<1.2 mmol/l]**	33	36.7	0	0.0	68.17	4.08; 139.41	0.003*
**Normal and high** **[≥1.2 mmol/l]**	57	63.3	58	100.0			
**Total**	90	100.0	58	100.0	---	---	---
**Mean ± SD**	68.1±18.7		129.2±32.6		---		0.001**

* Chi-square test was used, d.f=1; ** Independent T-test of two means was used

Higher serum TSH was found in 100% of the study group compared to only 6.9% in the control group (p<0.001). The mean serum TSH concentration in short stature group is 9.17±2.64 µUl/ml, which is significantly (p<0.001) higher than the level in the control group (2.28±1.10 µUl/ml), as presented in Table 4.

**Table 4:** Serum levels of TSH in short stature and control groups

TSH level	Cases “Short stature”		Control “Normal height”		Odd ratio	95% C.I.	P-value
	No.	%	No.	%			
**High** **[>4.20 µUl/ml]**	90	100.0	4	6.9	219.21	15.76; 415.09	0.001*
**Normal and low** **[≤4.20 µUl/ml]**	0	0.0	54	93.1			
**Total**	90	100.0	58	100.0	---	---	---
**Mean ± SD**	9.17±2.64		2.28±1.10		---		0.001**

* Chi-square test was used, d.f=1; ** Independent T-test of two means was used

**Figure 1:**
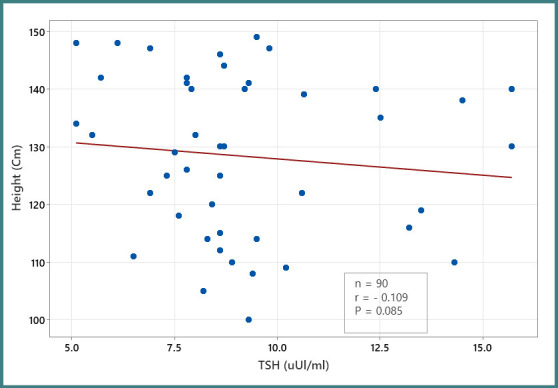
Correlation between serum TSH and children’s height in the short stature group [N=90]

Figure 1 shows that there was a statistically insignificant negative correlation of serum TSH with height (r = - 0.109, p <0.085).

## DISCUSSION

Short stature is a common feature of children with juvenile hypothyroidism once brain development is complete because thyroid hormones promote bone growth and skeletal development both directly and indirectly through their effects on the growth hormone. Juvenile hypothyroidism is caused by a lack of thyroid hormones as a result of the gland's destruction by autoimmune diseases [5-6]. When comparing the male and female genders, the latter had a significantly higher prevalence of short stature, which is consistent with the results of Gutch *et al*., where the female gender presented a double incidence of the condition [9].

In the current study, serum T4 concentrations were significantly lower and TSH concentrations were significantly higher in the study group compared to the control group.

Before growth hormone therapy for children with short stature, it is important to take into account the detrimental effects of thyroid hormone shortage on linear development when free T4 levels are lower than the normal range.

Treatment with levothyroxine for four months promotes an increase in growth velocity and a better response to growth hormone supplementation in short-stature children with low to normal thyroid hormone concentrations. The study should involve measures of both free thyroxin and thyroid stimulating hormone to differentiate between thyroid (primary) and central causes of hypothyroidism, (pituitary or even hypothalamic causes) because evaluation of thyroid stimulating hormone alone will not detect central hypothyroidism. Childhood growth delay is a well-known condition and may manifest with other symptoms resulting from hypothyroidism. Many children with hypothyroidism have a relatively normal development after diagnosis and treatment of hypothyroidism [12-14].

Increased blood TSH in the short stature group is consistent with the findings of Virmani *et al*., they reported that 45.45% of the children in the sample had impaired thyroid function, out of which 25.45% had high radioactive iodine uptake and raised TSH, while 20% had primary hypothyroidism with low or normal uptake [15]. This is crucial to distinguish from central hypothyroidism caused by TSH or TSH-releasing hormone (TRH) deficiency, which is usually associated with growth hormone deficiency (GHD) [16].

The T3 level differs insignificantly between the study and control group; in the case of short stature with chronic kidney disease, the total and free serum T3 and T4 levels are either normal or low [17].

## CONCLUSION

Juvenile hypothyroidism produces various skeletal manifestations such as short stature. Moreover, normal serum T3 with low serum thyroxin and high serum thyroid stimulating hormone is common in juvenile hypothyroidism.

## Personal thanks

The authors acknowledge the Department of Health of Nineveh/Al-Waffa Diabetic Centre for the great help in data collection. We also acknowledge assistant prof. Humam Alzubeer from the Department of Public Health, College of Medicine, University of Mosul for his support in conducting the statistical analysis.
